# Inclusion of interleukin-6 improved the performance of postoperative acute lung injury prediction for patients undergoing surgery for thoracic aortic disease

**DOI:** 10.3389/fcvm.2023.1093616

**Published:** 2023-08-11

**Authors:** Huili Li, Weiqi Feng, Qiuji Wang, Chenxi Li, Jiade Zhu, Tucheng Sun, Jinlin Wu

**Affiliations:** Department of Cardiac Surgery, Guangdong Cardiovascular Institute, Guangdong Provincial People’s Hospital, Guangdong Academy of Medical Sciences, Guangzhou, China

**Keywords:** interleukin-6, acute lung injury, thoracic aortic disease, postoperative, predict

## Abstract

**Background:**

We studied acute lung injury (ALI) in thoracic aortic disease (TAD) patients and investigated the predictive effect of interleukin-6 (IL-6) in acute lung injury after thoracic aortic disease.

**Methods:**

Data on 188 TAD patients, who underwent surgery between January 2016 to December 2021 at our hospital, were enrolled in. We analyzed acute lung injury using two patient groups. Patients with No-ALI were 65 and those with ALI were 123. Univariate logistic, LASSO binary logistic regression model and multivariable logistic regression analysis were performed for acute lung injury.

**Results:**

Preoperative IL-6 level was lower (15.80[3.10,43.30] vs. 47.70[21.40,91.60] pg/ml, *p* < 0.001) in No-ALI group than in ALI group. The cut-off points, determined by the ROC curve, were preoperative IL-6 > 18 pg/ml (area under the curve: AUC = 0.727). Univariate logistic regression analysis showed 19 features for TAD appeared to be early postoperative risk factors of acute lung injury. Using LASSO binary logistic regression, 19 features were reduced to 9 potential predictors (i.e., Scrpost + PLTpost + CPB > 182 min + D-dimerpost + D-dimerpre + Hypertension + Age > 58 years + IL6 > 18 pg/ml + IL6). Multivariable logistic regression analysis showed that Postoperative creatinine, CPB > 182 min and IL-6 > 18 pg/ml were early postoperative risk factors for ALI after TAD, and the odds ratios (ORs) of postoperative creatinine, CPB > 182 min and IL-6 > 18 pg/ml were 1.006 (1.002–1.01), 4.717 (1.306–19.294) and 2.96 (1.184–7.497), respectively. When postoperative creatinine, CPB > 182 min and IL-6 > 18 pg/ml (AUC = 0.819), the 95% confidence interval [CI] was 0.741 to 0.898. Correction curves were nearly diagonal, suggesting that the nomogram fit well. The DCA curve was then drawn to demonstrate clinical applicability. The DCA curve showed that the threshold probability of a patient is in the range of 30% to 90%.

**Conclusions:**

The inclusion of interleukin-6 demonstrated good performance in predicting ALI after TAD surgery.

## Introduction

Thoracic aortic disease (TAD) contains aneurysms and acute aortic syndromes (intramural hematomas, dissections, penetrating atherosclerotic ulcers). Thoracic aortic disease (TAD) is a type of vascular disease, which if left untreated, could cause fatal complications. Surgery is an important treatment modality for TAD. Aortic dissection is the worst complication of thoracic aortic disease (1). Acute aortic dissection is commonly reported to be accompanied by acute lung injury (ALI), where oxygenation of the lungs has been impaired severely (2). Acute lung injury (ALI) is a devastating and potentially life-threatening postoperative complication that can also prolong the duration of ventilator support and hospital stay. In large series of patients undergoing open surgery for thoracoabdominal aortic aneurysm, serious lung damage was reported at 60% (3, 4), and in recent publications, it was reported that it was between 40% and 50% (5). Acute hypoxic respiratory insufficiency caused by ALI is caused by a variety of factors that damage alveolar epithelial cells and capillary endothelial cells. Due to these pathological changes, ventilation is reduced, gas exchange is impaired, the ventilation-perfusion imbalance is severe, hypoxia is experienced, and pulmonary compliance is poor (6).

A significant role is played by inflammation in TAD development. A significant association between TAD and elevated plasma inflammatory markers has been reported (7, 8). On the one hand, the lung contains a considerable number of monocytes and macrophages in the cytoplasm to perform a protective function since the trachea is in direct touch with the outside environment; On the other hand, large and slow amount of circulating blood is needed to exchange gas between erythrocytes and alveoli. Lungs are therefore susceptible to inflammatory damage due to this feature (9). The role of inflammatory biomarkers as prognostic indicators after cardiac surgery has gained increasing attention. The impact of perioperative inflammatory biomarkers on clinical outcomes has been understudied in patients undergoing surgery for TAD.

There is a close correlation between cardiovascular disease and intercellular IL-6, which is an inflammatory cytokine that plays an important role in inflammation and immune response (10). The association between interleukin-6 (IL-6) and the pathology of aortic dissection has been demonstrated in numerous basic studies. As well, clinical studies have shown that elevated IL-6 can lead to complications after cardiovascular surgery (11–13). Therefore, to explore if IL-6 plays a predictive role in the occurrence of postoperative ALI in TAD patients undergoing surgery, we collected data on TAD patients from January 2016 to December 2021 who had IL-6 measurement.

## Methods

### Patient population

**Figure 1:**
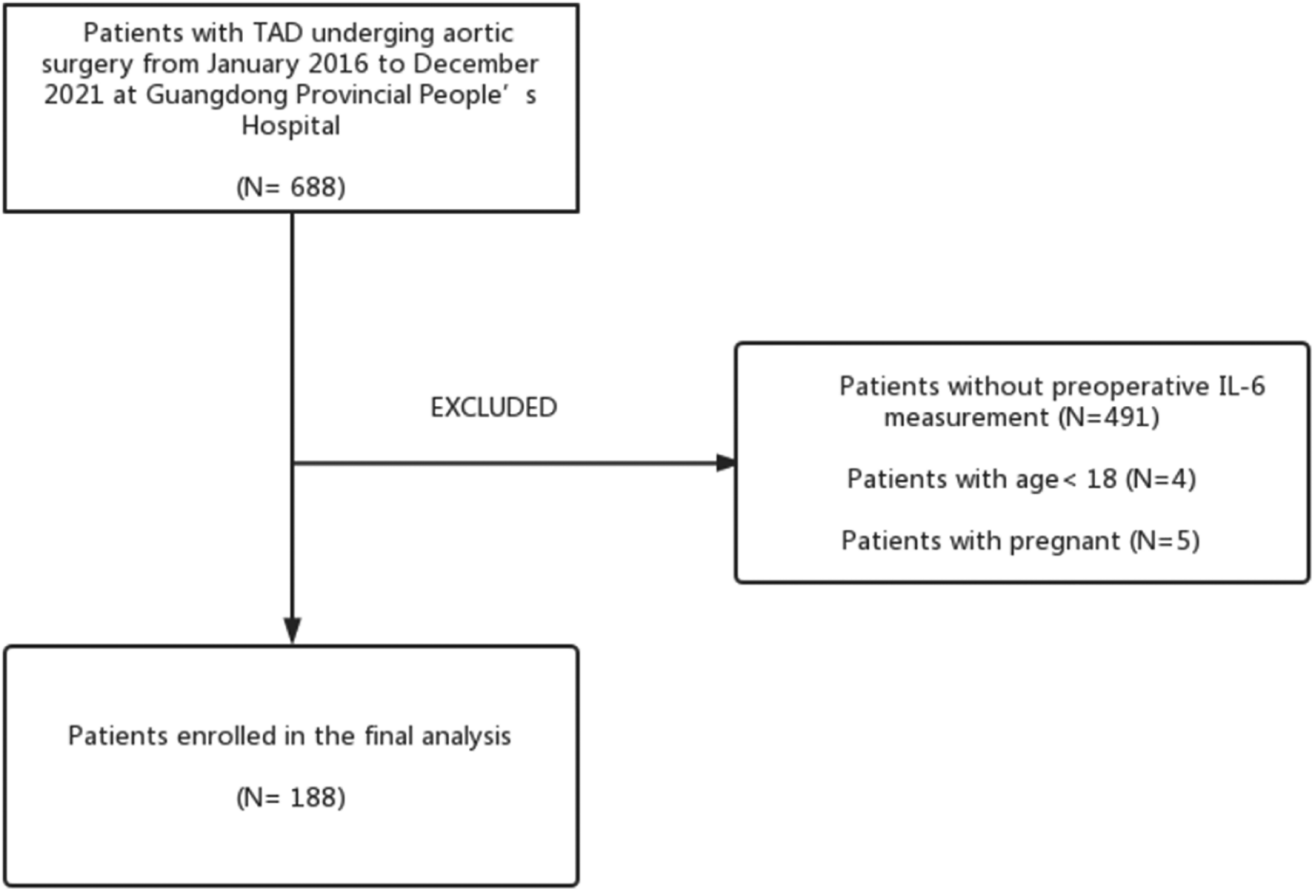
Patient screening chart.

The cohort of this retrospective study consisted of 188 patients with surgical treatment for TAD who had preoperative levels of IL-6 measured from Guangdong Provincial People's Hospital (Guangzhou, China) from January 2016 to December 2021. Figure 1 shows the patient screening process. ALI and Non-ALI are classified according to the presence or absence of acute lung injury after surgery. In our study, we included patients whose CT angiography or magnetic resonance angiography (MRA) confirmed the presence of TAD. The exclusion criteria were as follows: (1) No preoperative IL-6 measurement; (2) Age less than 18 years; (3) Death before surgery; (4) Malignant tumors. An overview of the clinical characteristics of the TAD patients included in the study can be found in Table 1. This study protocol was approved by the Institutional Ethics Committee of Guangdong Provincial People's Hospital (KY-Q-2021-183-02) and conformed to the Declaration of Helsinki.

**Table 1:** Preoperative data.

Variables	All (*n* = 188)	No-ALI (*n* = 65)	ALI (*n* = 123)	*p*
Age, mean (±SD), years	54.31 ± 12.02	50.97 ± 11.49	56.07 ± 11.92	0.005
Male, *n* (%)	142 (75.53)	54 (83.08)	88 (71.54)	0.080
BMI, mean (±SD), kg/m^2^	24.34 ± 3.79	23.62 ± 3.33	24.72 ± 3.96	0.069
Atria fibrillation, *n* (%)	10 (5.75)	4 (6.25)	6 (5.45)	0.828
Hypertension, *n* (%)	131 (69.68)	38 (58.46)	93 (75.61)	0.015
Diabetes mellitus, *n* (%)	7 (3.72)	3 (4.62)	4 (3.25)	0.639
Hyperlipidemia, *n* (%)	63 (34.62)	19 (29.23)	44 (37.61)	0.255
Chronic kidney disease, *n* (%)	20 (10.64)	4 (6.15)	16 (13.01)	0.147
Smoking, *n* (%)	36 (19.25)	9 (14.06)	27 (21.95)	0.194
Cardiac aortic surgery, *n* (%)	11 (5.85)	4 (6.15)	7 (5.69)	0.898
COPD, *n* (%)	32 (17.20)	14 (21.88)	18 (14.75)	0.222
BAV, *n* (%)	7 (3.74)	4 (6.15)	3 (2.46)	0.205
MFS, *n* (%)	8 (4.28)	5 (7.69)	3 (2.46)	0.092
Shock hypotension, *n* (%)	2 (1.06)	0 (0.00)	2 (1.63)	0.301
AI greater than 2, *n* (%)	28 (46.67)	10 (43.48)	18 (48.65)	0.696
Pleural Effusion, *n* (%)	27 (14.36)	6 (9.23)	21 (17.07)	0.145
Cardiac Effusion, *n* (%)	50 (26.60)	9 (13.85)	41 (33.33)	0.004
Renal Cyst, *n* (%)	65 (35.14)	19 (30.16)	46 (37.70)	0.308
Liver cyst, *n* (%)	55 (29.73)	19 (30.16)	36 (29.51)	0.927
Preoperative condition				
IL6, median [IQR], pg/ml	39.30[10.60,74.20]	15.80[3.10,43.30]	47.70[21.40,91.60]	<0.001
WBC, mean (±SD), 10^9^/L	10.74 ± 3.92	9.61 ± 4.10	11.31 ± 3.69	0.005
PLT, mean (±SD), 10^9^/L	212.32 ± 104.96	220.98 ± 105.36	207.89 ± 104.48	0.424
D-dimer, mean (±SD), ng/ml	8,121.19 ± 7,824.67	4,848.88 ± 6,284.89	9,730.52 ± 8,003.38	<0.001
glucose, mean (±SD), mg/dl	7.07 ± 2.10	6.58 ± 2.21	7.32 ± 2.00	0.023
AST, mean (±SD), U/L	78.55 ± 376.31	24.34 ± 13.61	105.44 ± 457.79	0.171
ALT, mean (±SD), U/L	61.68 ± 245.19	21.88 ± 16.22	81.08 ± 296.89	0.133
LDL, mean (±SD), mmol/L	2.93 ± 0.77	2.85 ± 0.76	2.97 ± 0.77	0.339
Tirglyceride, mean (±SD), mh/dl	1.33 ± 0.58	1.28 ± 0.56	1.36 ± 0.59	0.426
Scr, mean (±SD), umoI/L	103.45 ± 68.32	86.50 ± 25.61	112.13 ± 80.63	0.002
Maximum Diameter, median [IQR], mm	48.00[42.00,57.00]	50.00[41.00,59.00]	48.00[43.00,57.00]	0.877

### Definitions

ALI severity was put into the following categories based on the Berlin definition: mild ALI [200 mmHg < oxygen index (OI) ≤ 300 mmHg], moderate ALI (100 mmHg < OI ≤ 200 mmHg), and severe ALI (OI ≤ 100 mmHg) (14). Early mortality was defined as all-cause mortality in-hospital. Stroke was defined as a permanent neurologic injury with clinical or radiographic evidence, such as CT or MRI. The term MODS refers to the acute and potentially reversible dysfunction of two or more organ systems resulting from a variety of clinical factors (15).

### Data collection

Before surgery, venous blood was extracted from all patients (within 48 h of the onset of symptoms). The preoperative indices of all patients were observed and summarized in Table 1. Preoperative indicators included age, gender, body mass index (BMI), IL-6, D-dimer, serum creatinine (SCR), triglyceride, low density lipoprotein (LDL), alanine aminotransferase (ALT), aspartic transaminase (AST), glucose, white blood cells (WBC), platelets (PLT), cardiac effusion, hypertension, and other previous history. Intraoperative and postoperative indicators were summarized in Table 2. Intraoperative and postoperative indicators included cardiopulmonary bypass (CPB), aortic cross-clamp time (ACC), deep hypothermic circulation arrest (DHCA), WBC, PLT, D-dimer, glucose, SCR, AST, ALT, mediastinitis, and other postoperative complications.

**Table 2:** Surgical data.

Variables	All (*n* = 188)	No-ALI (*n* = 65)	ALI (*n* = 123)	*p*
Elective surgery, *n* (%)	98 (52.128)	40 (62.500)	58 (46.774)	0.041
Concomitant procedures, *n*%				
Bentall, *n* (%)	55 (29.255)	25 (39.063)	30 (24.194)	0.034
Cabrol, *n* (%)	6 (3.191)	2 (3.125)	4 (3.226)	0.970
Wheats, *n* (%)	8 (4.255)	6 (9.375)	2 (1.613)	0.012
FET, *n* (%)	160 (85.106)	47 (73.438)	113 (91.129)	0.001
Operation details				
CPB time, median [IQR], min	232.00[198.00,263.00]	227.00[182.00,245.00]	233.00[202.00,266.00]	0.045
ACC time, median [IQR], min	119.00[91.00,146.00]	117.00[87.00,146.00]	119.00[94.00,146.00]	0.520
DHCA time, mean (±SD) min,	16.84 ± 8.86	15.84 ± 9.48	17.38 ± 8.46	0.260
DHCA temp nasal, median [IQR], °C	24.00[22.20,25.00]	24.00[22.50,25.30]	23.80[22.10,25.00]	0.221
CPB > 187.5 min, *n* (%)	160 (85.11)	47 (72.31)	113 (91.87)	<0.001
ACC > 172.5 min, *n* (%)	25 (13.30)	4 (6.15)	21 (17.07)	0.036
DHCA, *n* (%)	164 (87.23)	52 (80.00)	113 (91.87)	0.018
RBC transfusionU, median [IQR]	5.000[1.500,8.000]	4.000[1.000,6.000]	5.500[3.500,10.000]	0.008

### Statistical analysis

Continuous variables were reported with mean ± standard deviation (SD) and were compared using Student's independent *t*-test. If normality was not assumed (Kolmogorov-Smirnov test), median (interquartile range, IQR) and Mann-Whitney *U* test would be used. Categorical variables were presented as numbers and percentages and were compared using the Chi-square test or Fisher's exact test (if expected value ≤5 was found). Univariate and multivariate logistic regression models and estimated odds ratio (OR) were used to investigate the association between independent variables and in-hospital mortality. Partial nonbinary variables were dichotomized via univariable logistic regression and optimal cut-off points were estimated via receiver operating characteristic (ROC) curve analysis and determined based on the maximum Youden index.

We selected the most useful predictive features from the primary data set by using the least absolute shrinkage and selection operator (LASSO) method. This method is suitable for reducing high-dimensional data. With the LASSO binary logistic regression model, all the clinicopathologic variables in the cohort were reduced to a limited set of potential predictors. If the penalization coefficient lambda (*λ*) is high, the predicted regression parameters are unaffected; but, as the coefficients decrease, certain coefficients may be reduced to zero. We then selected the optimal *λ* in the LASSO model by using 10-fold cross-validation via minimum criteria and one standard error of the minimum criteria (the 1-SE criterion). Lastly, the Lasso method was used to select all coefficients that were not zero (Figure 1) to re-fit the model. The variables that were also significant in multivariate results would be recognized as associated factors in patients' ALI. A *p* < 0.05 would be recognized as reaching significance for each test, two-tailed. All analyses were performed using IBM SPSS Version 25 (SPSS Statistics V25, IBM Corporation, Somers, New York) and R software (version 4.2.1).

The model was assessed using the area under the ROC curve. The AUC (area under the receiver operating characteristic curve) was measured to quantify the discrimination performance of the model. Based on the cohort, we conducted decision curve analyses to determine the clinical usefulness of the model. This model's calibration was assessed using calibration plots.

## Results

### Clinical characteristics

In this study, 188 patients undergoing TAD surgery were included after inclusion and exclusion criteria were met. The mean age of patients was 54.31 ± 12.02 years. In addition, 75.53% of patients were male. According to the postoperative OI, ALI was identified in 123 patients and the incidence of preoperative ALI was 65.4%. As shown in Table 1, patients who have and do not have postoperative ALI are compared in terms of their basic characteristics. The ALI patients were older (56.07 ± 11.92 vs. 50.97 ± 11.49; *p* = 0.005), had a higher IL6 (15.8 VS. 47.7; *p* < 0.001), a higher WBC (9.61 ± 4.10 VS. 11.31 ± 3.69; *p* = 0.005), a higher D-dimer (4,848.88 ± 6,284.89 vs. 9,730.52 ± 8,003.38; *p* < 0.001), a higher glucose (6.58 ± 2.21 vs. 7.32 ± 2.00; *p* = 0.023) and SCR (86.50 ± 25.61 vs. 112.13 ± 80.63; *p* = 0.002). Compared to no-ALI, a greater percentage of ALI were hypertensive (75.61% vs. 58.46%; *p* = 0.015). There was no statistically significant difference between the ALI and no-ALI groups in other factors. Patients with lung injury have a relatively worse postoperative situation (Table 3).

**Table 3:** Postoperative data.

Variables	All (*n* = 188)	No-ALI (*n* = 65)	ALI (*n* = 123)	*p*
Postoperative condition				
SCR > 200.10 umoI/L, *n* (%)	64 (34.0)	9 (13.80)	55 (44.72)	<0.001
Early Mortality, *n* (%)	18 (9.57)	2 (3.08)	16 (13.01)	0.028
Stroke, *n* (%)	17 (9.04)	3 (4.62)	14 (11.38)	0.124
Delirium, *n* (%)	35 (18.62)	5 (7.69)	30 (24.39)	0.005
Paresis, *n* (%)	5 (2.66)	0 (0.00)	5 (4.07)	0.099
CRRT, *n* (%)	37 (19.68)	0 (0.00)	37 (30.08)	<0.001
Re-exploration for bleeding delayed chest closure, *n* (%)	10 (5.32)	1 (1.54)	9 (7.32)	0.093
Tracheotomy, *n* (%)	9 (4.79)	0 (0.00)	9 (7.32)	0.029
MODS, *n* (%)	25 (13.30)	1 (1.54)	24 (19.51)	<0.001
ECMO, *n* (%)	8 (4.26)	0 (0.00)	8 (6.50)	0.052
Mediastinitis, *n* (%)	3 (1.60)	1 (1.54)	2 (1.63)	0.964
Acute Liver Injury, *n* (%)	74 (39.36)	18 (27.69)	56 (45.53)	0.017
Post-op Hospital stay, median [IQR], day	21.00[16.00,31.00]	18.00[14.00,27.00]	23.00[18.00,39.00]	<0.001
ICU stay, median [IQR], day	89.00[14.00,186.00]	43.00[7.00,80.00]	148.00[21.00,257.00]	<0.001
Ventilation time, mean (±SD), h	159.55 ± 236.03	48.22 ± 44.96	217.48 ± 271.74	<0.001
WBC, mean (±SD), 10^9^/L	19.14[15.97,22.57]	18.03[15.67,20.44]	19.54[16.37,23.17]	0.089
PLT, mean (±SD), 10^9^/L	103.00[56.00,128.00]	116.00[79.00,147.00]	94.00[50.00,120.00]	0.003
D-dimer, mean (±SD), ng/ml	9,635.20 ± 6,717.40	7,694.63 ± 6,691.10	10,628.05 ± 6,510.87	0.018
Glucose, mean (±SD), mg/dl	12.04[9.38,14.27]	11.95[9.63,14.13]	12.04[9.25,14.33]	0.899
AST, mean (±SD), U/L	403.12 ± 1,911.01	106.60 ± 190.18	562.41 ± 2,349.84	0.122
Scr, mean (±SD), umoI/L	160.96[109.60,259.50]	124.10[95.90,172.36]	179.60[121.60,360.20]	<0.001
ALT, mean (±SD),U/L	62.00[29.00,117.00]	40.00[26.00,88.00]	70.00[30.00,152.00]	0.023

### Feature selection

**Figure 2:**
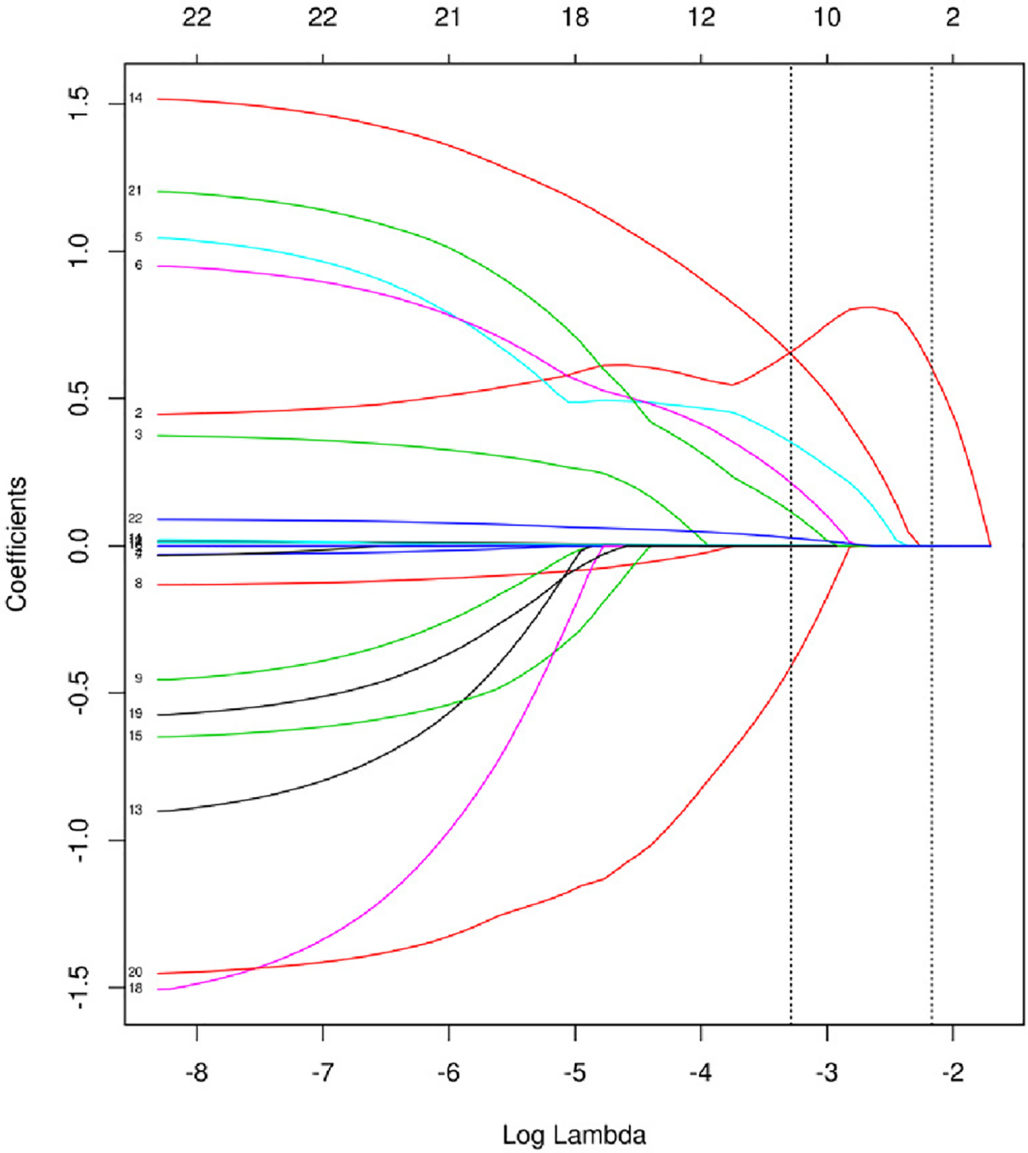
Texture feature selection using the least absolute shrinkage and selection operator (LASSO) binary logistic regression model.

**Figure 3:**
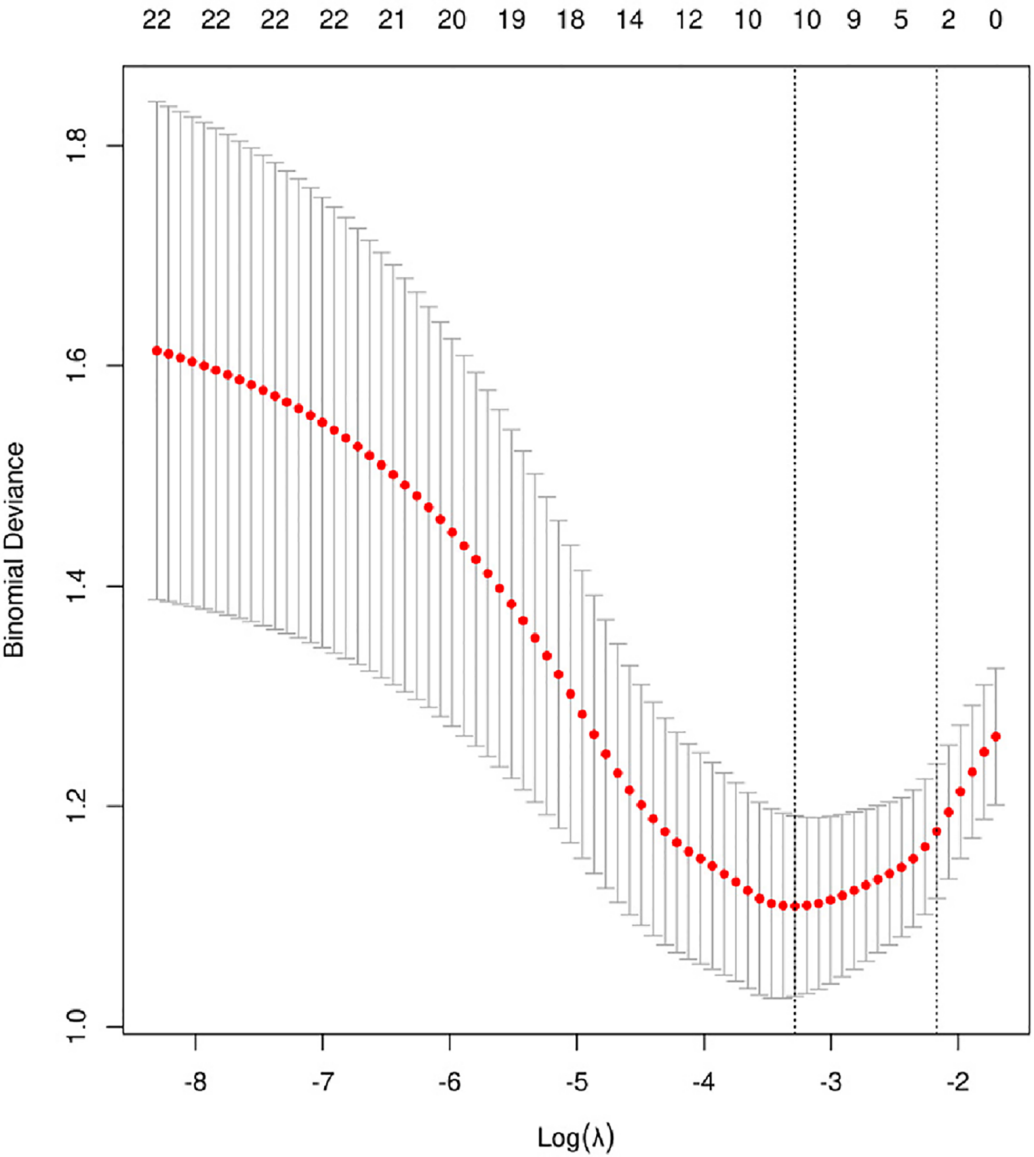
Cross-validation of the LASSO regression model. Lasso coefficient profiles of the 24 features. A coefficient profile plot was produced against the log (*λ*) sequence. The vertical line was drawn at the value selected using 10-fold cross-validation, where optimal resulted in 10 features with non-zero coefficients.

**Figure 4:**
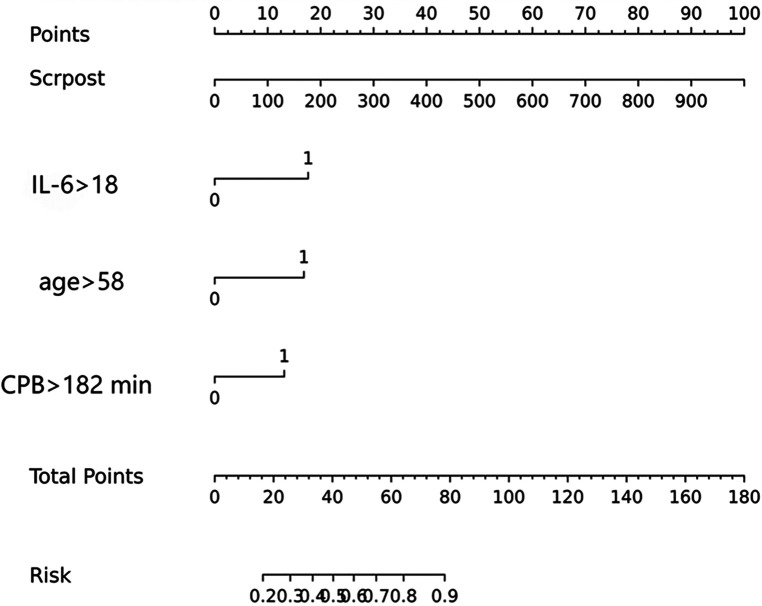
Nomogram to predict ALI in patients with TAD.

**Figure 5:**
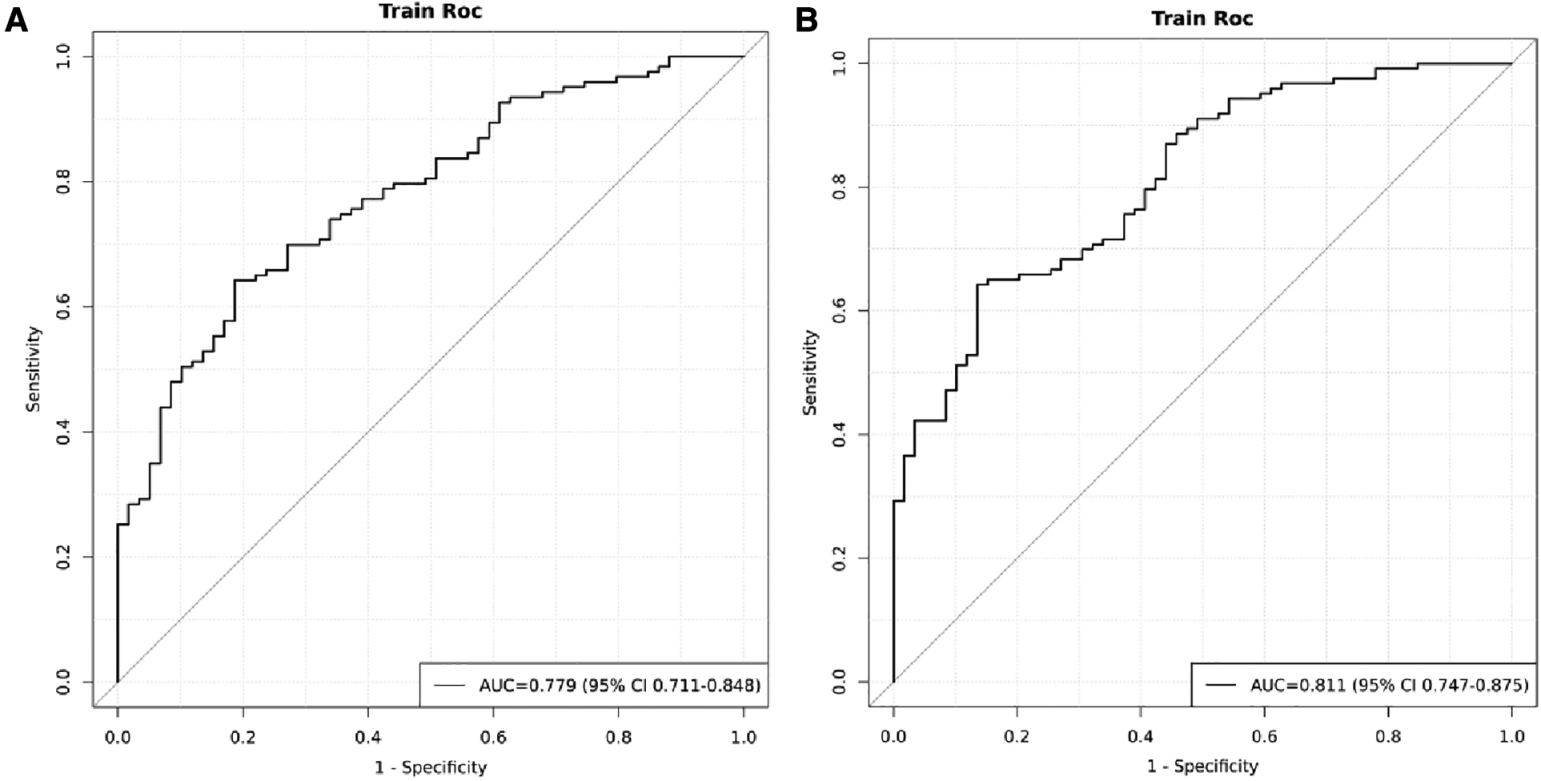
ROC of the cohort. (**A**) ROC curves when IL > 18 was excluded. (**B**) ROC curves at inclusion of postoperative creatinine, age > 58 and IL-6 > 18 pg/ml.

The model was built using LASSO binary logistic regression because the sample size in this study was insufficient to satisfy the advised number of events per variable. The *λ* value was 0.034. Of all the relevant variables, 24 features were reduced to 10 potential predictors on the basis of the cohort. The 10 variables with non-zero coefficients in the LASSO logistic regression model (i.e., Scrpost + RBC transfusionU + CPB > 182mi + D-dimerpre + Hypertension + Age > 58 years + Wheats + FET + IL6 > 18 pg/ml + IL6) were used in the final model (in Figures 2, 3). Next, the above mentioned 10 variables were included in the multivariable logistic regression analysis to determine the risk factors associated with postoperative ALI. Finally, postoperative creatinine, age > 58 and IL-6 > 18 pg/ml were as ALI risk factors (Tables 4, 5 and Figure 4). And ROC curve was shown in Figure 5.

**Table 4:** Univariate analyses for postoperative acute lung injury.

Variables	OR	95%CI	*p*-value
Preoperative condition			
IL6, pg/ml	1.02	[1.010,1.031]	0.000
Age, years	1.04	[1.010,1.064]	0.007
BMI, kg/m^2^	1.08	[0.993,1.179]	0.071
Age > 58 years	3.57	[1.738,7.342]	0.001
Male	0.51	[0.240,1.092]	0.083
Atria_fibrillation	0.87	[0.235,3.189]	0.828
Hypertension	2.20	[1.159,4.187]	0.016
Diabetes mellitus	0.70	[0.151,3.202]	0.640
Hyperlipidemia	1.46	[0.760,2.802]	0.256
Chronic kidney disease	2.28	[0.729,7.129]	0.156
Smoking	1.72	[0.754,3.918]	0.198
Hx of cardiac aortic surgery	0.92	[0.259,3.267]	0.898
COPD	0.62	[0.285,1.343]	0.224
Pleural Effusion	2.03	[0.773,5.299]	0.151
Cardiac Effusion	3.111	[1.401,6.906]	0.005
Renal Cyst	1.402	[0.731,2.687]	0.309
Liver cyst	0.969	[0.499,1.883]	0.927
WBC, 10^9^/L	1.127	[1.035,1.227]	0.006
PLT, 10^9^/L	0.999	[0.996,1.002]	0.423
D-dimer, ng/ml	1.000	[1.000,1.000]	0.000
Glucose, mg/dl	1.205	[1.023,1.420]	0.025
AST, U/L	1.018	[0.997,1.039]	0.089
ALT, U/L	1.008	[0.994,1.022]	0.258
LDL, mmol/L	1.235	[0.802,1.901]	0.337
SCR, umoI/L	1.010	[1.002,1.019]	0.017
WBC > 9.27 10^9^/L	2.651	[1.375,5.110]	0.004
SCR > 95.28 umoI/L	3.065	[1.331,7.060]	0.008
IL > 18 pg/ml	5.517	[2.850,10.679]	0.000
Elective surgery, *n* (%)	0.527	[0.285,0.977]	0.042
Concomitant procedures, *n*%			
Bentall, *n* (%)	0.498	[0.260,0.953]	0.035
Wheats, *n* (%)	0.158	[0.031,0.809]	0.027
FET, *n* (%)	3.716	[1.618,8.532]	0.002
Operation details			
CPB time, min	1.005	[1.000,1.011]	0.052
ACC time, min	1.003	[0.995,1.010]	0.472
DHCA time, min	1.020	[0.986,1.055]	0.259
DHCA temp nasal, °C	0.938	[0.851,1.034]	0.201
Ventilation time, min	1.019	[1.011,1.027]	0.000
CPB > 182 min	4.328	[1.860,10.070]	0.001
Acc > 172.5 min	3.140	[1.029,9.578]	0.044
DHCA	2.880	[1.185,7.002]	0.020
RBC transfusionU, median [IQR]	1.124	[1.041,1.213]	0.003
Postoperative condition			
RBC, 10^9^/L	0.194	[0.064,0.587]	0.004
WB, 10^9^/L	0.994	[0.975,1.013]	0.510
PLT, 10^9^/L	0.993	[0.988,0.998]	0.007
D-dimer, ng/ml	1.000	[1.000,1.000]	0.020
Glucose, mg/dl	0.975	[0.912,1.042]	0.457
AST, U/L	1.001	[1.000,1.003]	0.139
Scr,umoI/L	1.007	[1.004,1.011]	0.000
ALT, U/L	1.002	[0.999,1.005]	0.117
Scr > 200 umoI/L	5.033	[2.287,11.073]	0.000

**Table 5:** Multivariate analyses for postoperative acute lung injury.

Variables	OR (95% CI)	*p*
Postoperative serum creatinine	1.006 (1.002–1.01)	0.003
CPB > 182 min	2.141 (0.762–6.26)	0.152
Age > 58 years	2.654 (1.171–6.379)	0.023
IL6 > 18 pg/ml	2.781 (0.996–7.54)	0.048
IL	1.009 (0.999–1.025)	0.177

### Nomogram construction

The above-mentioned variables were screened using logistic regression backwards selection. Postoperative creatinine, age > 58 and IL-6 > 18 pg/ml were identified as ALI risk factors. The ALI risk was 2.78-fold higher in IL > 18 than in IL < 18 patients (95% CI = 1.0–7.54; *p* = 0.048), postoperative creatinine (OR for ALI: 1.006, 95% CI: 1.002–1.01, *p* = 0.005) and Age > 58 years (OR for ALI: 2.654, 95% CI: 1.171–6.379, *p* = 0.023) were independent risk factors for postoperative ALI (Table 4). These results were used to construct a nomogram for estimating the postoperative ALI risk in TAD patients during hospitalization (Figure 4). Three independent predictors of postoperative ALI were used. The three factors used to estimate the postoperative risk of ALI each received a score based on their value, and the total score was calculated by summing the scores.

### Nomogram validation

**Figure 6:**
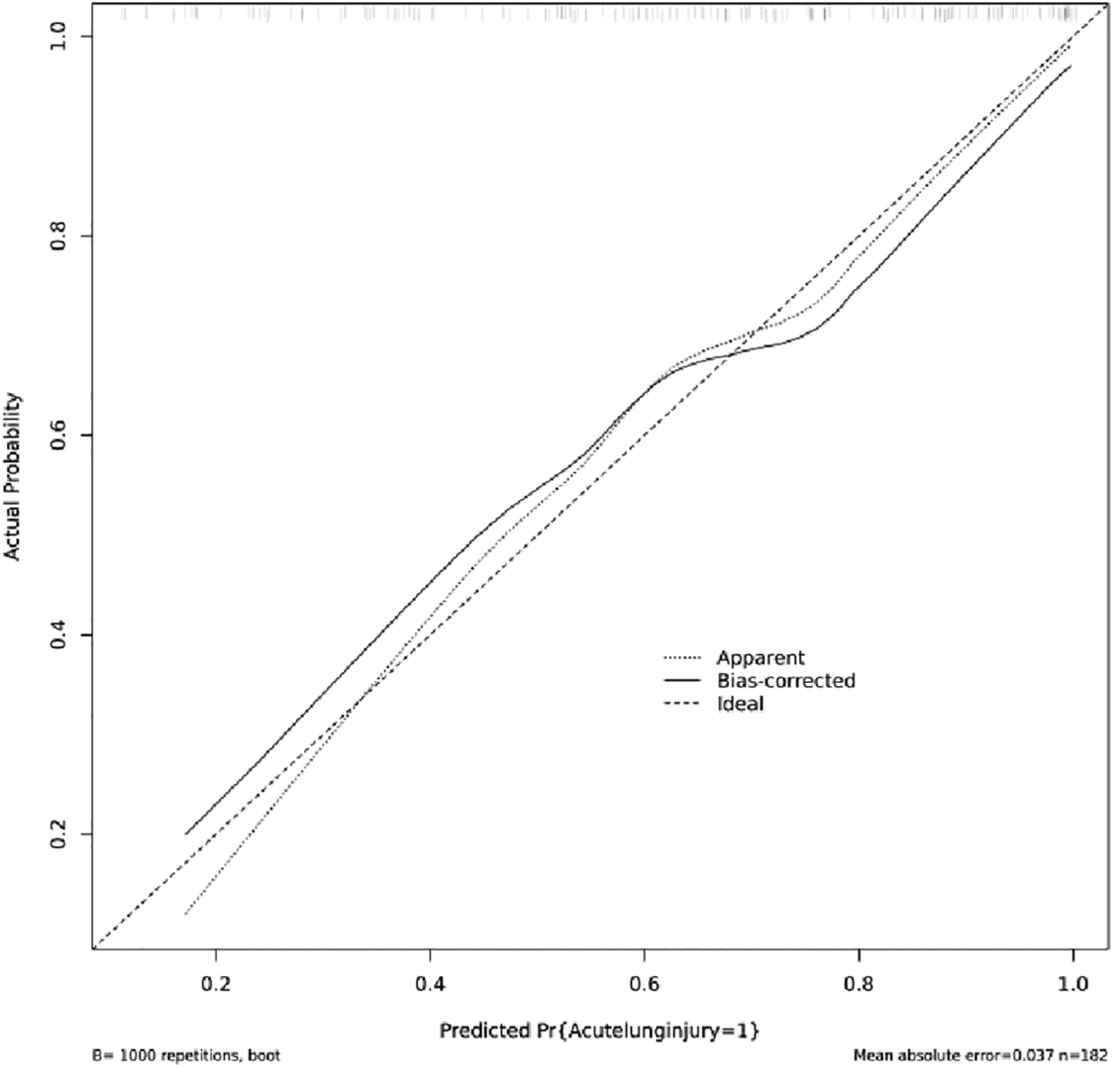
A calibration plot based on the nomogram.

**Figure 7:**
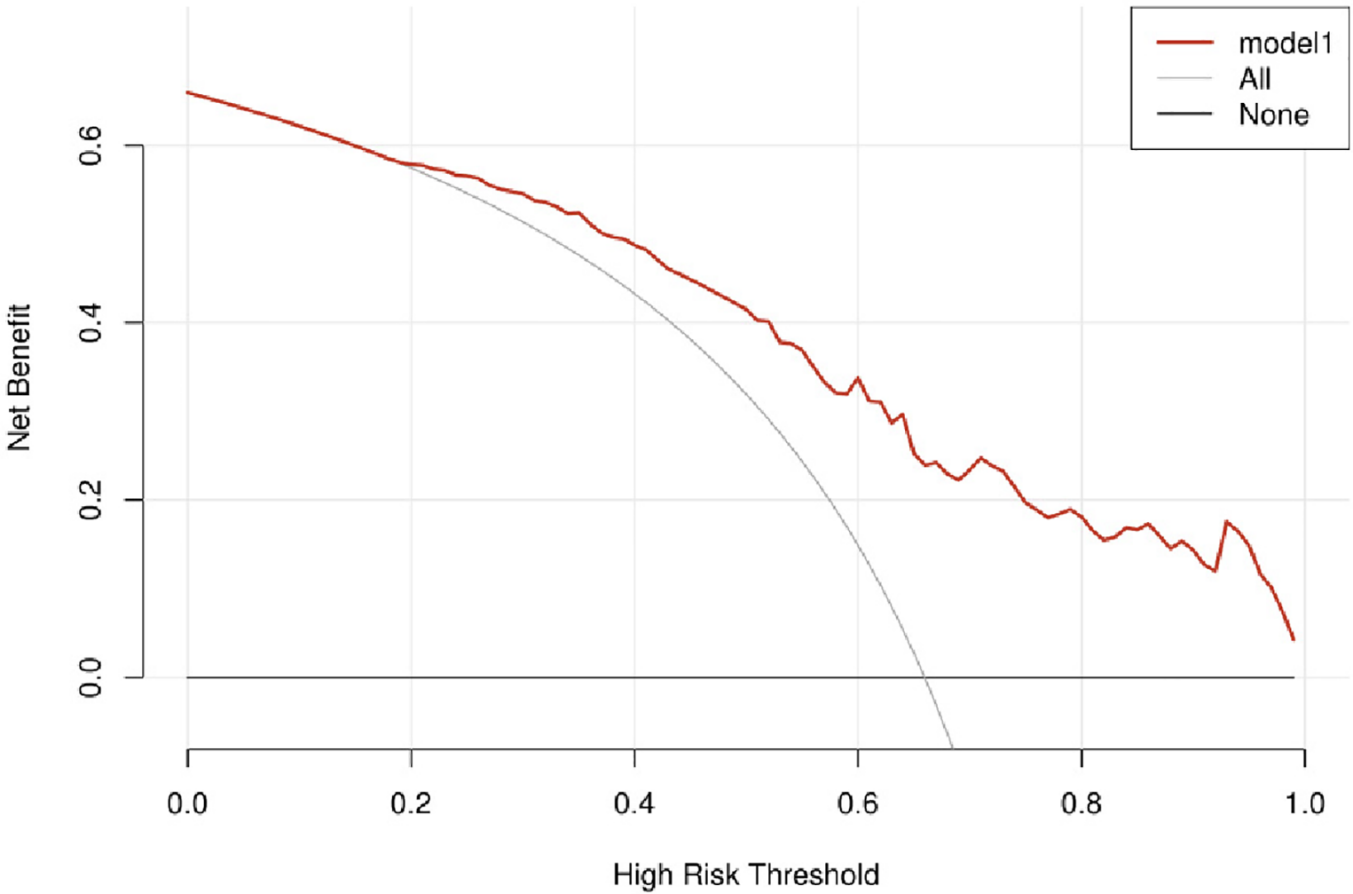
Decision curve.

Figure 4 indicated that the AUC values of the nomogram were 0.811(95% CI = 0.747–0.875) for the cohort. Figure 6 displayed the calibration curves of the nomogram. The Hosmer–Lemeshowchi-square statistic was 4.24 and the *p* value was 0.8348, demonstrating good calibration. In the cohort, correction curves were nearly diagonal, suggesting that the nomogram fit well. The DCA curve was then drawn to demonstrate clinical applicability (Figure 7). The DCA curve showed that the threshold probability of a patient is in the range of 30% to 90%, the use of a nomogram to predict postoperative ALI in patients with TAD is more beneficial than either the treat-all-patients or treat-none scheme.

## Discussion

We looked into whether adding IL-6 could predict occurrence of ALI after TAD surgery. Association of interleukin-6 with postoperative complications is an area of ongoing research. This study explores the predictive role of interleukin-6 in the occurrence of postoperative ALI after surgery for TAD and demonstrates that the inclusion of interleukin-6 improves the performance of predictive model for ALI after TAD surgery. We developed a diagnostic nomogram for the postoperative individualized prediction of ALI in patients with TAD during hospitalization. The nomogram incorporated three items: IL-6 > 18 pg/ml, ventilation time and postoperative serum creatinine. We selected an optimum IL-6 cutoff value based on the Youden index (Youden index = sensitivity + specificity-100%) for the prediction of patients with postoperative ALI, and also considered the sensitivity and specificity. In practice, the choice of cutoff value might be dependent on the risk of misdiagnosis and missed diagnosis. Our study indicated that IL-6 > 18 pg/ml, ventilation time and postoperative serum creatinine are independent risk factors for postoperative ALI in TAD patients. The validity of our nomogram model was determined using multiple indicators, including AUC, correction curve, Hosmer-Lemeshow test and DCA. Through shrinking the regression coefficients that represent the correlation between predictor and outcome, 24 candidate features were reduced to 10 potential predictors for the construction of the model. This strategy outperforms the method of selecting predictors based on the strength of their univariable correlation with the result.

A number of studies found that blood or lung tissues from aortic dissection and thoracic aortic aneurysm patients with postoperative ALI showed significant increases in C-reactive protein (CRP), interleukin-6 (IL-6), and monocyte chemoattractant protein-1 (MCP-1). As a result of these reactions, pulmonary microvascular endothelial cells (PMVECs) are subject to apoptosis and barrier dysfunction, which lead to the elevation of endothelial permeability and the development of ALI (5, 16). Patients with ALI had higher levels of blood IL-6. Experimental evidence suggested aortic aneurysms can secrete IL-6 (17–19). In basic experimental research, Paige et al. found aortic rupture and death can be decreased in AAA mouse models by selectively inhibiting the IL-6 trans-signaling pathway, which suggested a possible therapeutic target (20). The expression of IL-6 was increased in AD rat models. MMP-2 expression may be enhanced by IL-6, which may promote extracellular matrix degradation, which promoted AD development (19). In clinical studies, the IL-6 level was found to be elevated in postoperative delirium (POD) patients following aortic dissection surgery by Lv Xiaochai et al. In this way, plasma IL-6 levels could be used to evaluate the outcomes of POD in AAD patients (21). Furthermore, high levels of IL-6 and D-dimer have predictive significance in predicting poor prognosis following acute Stanford type A aortic dissection surgery (13). Researchers have looked into the link between IL-6 (22), but it is not clear if IL-6 also affects ALI after TAD surgery.

In our study, patients with postoperative ALI had higher levels of IL-6 than those who did not have ALI, and the difference was statistically significant. After using logistic regression, it was found that IL-6 > 18 was a risk factor for postoperative ALI. Moreover, the AUC in the development cohort was 0.811 and correction curves were nearly diagonal. Compared with other studies, we selected the cutoff value, but whether this cutoff value can be applied to the subsequent validation still needs to be explored by expanding the sample size.

In cardiac surgery, a serious problem associated with cardiopulmonary bypass (CPB) was that it triggers an inflammatory response that contributed to the dysfunction of various organs, including the lungs (23, 24). The complement system in the blood and many different body cells were broadly stimulated in the particular cardiopulmonary bypass environment, and their functions were progressively damaged. During the postoperative period, their own inflammatory response was amplified, leading to SIRS and further causing organ dysfunction, including ALI. Lung immunological and anatomical characteristics contribute directly to the poor prognosis of ALI, which makes it one of the main targets of inflammation (25). A prolonged cardiopulmonary bypass will, therefore, result in postoperative ALI in ATAAD surgery.

The kidneys were responsible for eliminating creatinine from the body. Increased creatinine levels suggest renal disease. However, a statistically significant difference was found in postoperative serum creatinine levels higher in the ALI group in our study. Despite being different organs with their own location, structure, and function, lung and kidney tissue could be damaged simultaneously in the course of systemic diseases since they were not completely independent from one another (26). It has been proposed that renal ischemia/reperfusion could cause the systemic release of injurious factors that activated pulmonary capillary endothelial cells, resulting in increasing permeability and expression of adhesion molecules in the cells (27). Previous studies have found in the univariate analysis, increased creatinine levels were a significant risk factor for hypoxemia at 1 and 12 h after surgery (28). A possible explanation was that this group of patients was more susceptible to fluid overload during surgery due to their increased sensitivity to fluids. Some investigators have identified excessive hemodilution during CPB as an important factor in the development of lung injury (28). We were unable to determine hemodilution and fluid load because fluid intake and output were not counted in our data.

The shortcoming of our study is that relatively few previous tests for IL-6 have been performed, so the number of patients included in the study is relatively insufficient, which may make the results less perfect. Therefore, for follow-up studies, preoperative testing of IL-6 in patients should be increased. IL-6 testing is not included as a mandatory preoperative test, but since studies have found that IL-6 has a predictive effect on lung injury, it should be recommended that patients be tested for IL-6 prior to surgery in order to better prevent postoperative lung injury. We also hope to explore the cut-off value of IL-6 in a follow-up study.

## Data Availability

The original contributions presented in the study are included in the article/Supplementary Material, further inquiries can be directed to the corresponding authors.
