# Supplementary material for: Inclusion of interleukin-6 improved the performance of postoperative acute lung injury prediction for patients undergoing surgery for thoracic aortic disease
Source: Front Cardiovasc Med. 2023 Aug 11;10:1093616. doi: 10.3389/fcvm.2023.1093616 (PMC10457658; doi:10.3389/fcvm.2023.1093616)
Supplement: Supplementary file 1 [file Datasheet1.pdf]

Abbreviations and Acronyms

TAD=thoracic aortic disease
ALI= acute lung injury
IL-6=interleukin-6
MRA=magnetic resonance angiography
OI=oxygen index
BMI=body mass index
SCR=serum creatinine
LDL=low density lipoprotein
ALT=alanine aminotransferase
AST=aspartic transaminase
WBC=white blood cells
CPB=cardiopulmonary bypass
ACC=aortic cross-clamp time
DHCA=deep hypothermic circulation arrest
OR=odds ratio
ROC=receiver operating characteristic
LASSO=least absolute shrinkage and selection operator
AUC=area under the receiver operating characteristic curve
CRP=C-reactive protein
MCP-1=monocyte chemoattractant protein-1
PMVECs=pulmonary microvascular endothelial cells
[illegible]
